# MicroRNAs during *Echinococcus* spp. infection: a comprehensive systematic review and implications for diagnosis and therapy

**DOI:** 10.1186/s13071-026-07581-0

**Published:** 2026-07-23

**Authors:** Maura Rojas-Pirela, Diego Andrade-Alviárez, Verónica Rojas, Christian Castillo, Alejandro Prieto-Rojas, Vanessa Rueda-Cala, Cristina Carbonell, Daniel Salete-Granado, Candy Pérez, María A. Pérez-Nieto, Paul A. M. Michels, Wilfredo Quiñones, Miguel Marcos, Ulrike Kemmerling

**Affiliations:** 1 Instituto de Investigación Biomédica de Salamanca (IBSAL), 37007 Salamanca, Spain; 2 Departamento de Medicina, Universidad de Salamanca, 37007 Salamanca, Spain; 3 Departamento de Medicina Interna, Hospital Universitario de Salamanca, 37007 Salamanca, Spain; 4 Núcleo Interdisciplinario de Biología y Genética, Instituto de Ciencias Biomédicas (ICBM), Facultad de Medicina, Universidad de Chile, Independencia 1027, Santiago de Chile, Chile; 5 Laboratorio de Enzimología de Parásitos, Departamento de Biología, Facultad de Ciencias, Universidad de Los Andes, Mérida, 5101 Venezuela; 6 Instituto de Biología, Facultad de Ciencias, Pontificia Universidad Católica de Valparaíso, 2373223 Valparaíso, Chile; 7 Facultad de Medicina, Universidad de Los Andes, Mérida, 5101 Venezuela; 8 Departamento de Enfermedades Infecciosas, Hospital Universitario de Salamanca, 37007 Salamanca, Spain; 9 Centro de Investigación de Enfermedades Tropicales de la Universidad de Salamanca (CIETUS), 37007 Salamanca, Spain; 10 Fundación Instituto de Estudios de Ciencias de La Salud de Castilla y León, 42002 Soria, Spain; 11 School of Biological Sciences, University of Edinburgh, The King’s Buildings, Edinburgh, EH9 3FL UK; 12 Faculty of Applied Sciences, UCSI University, Kuala Lumpur, Malaysia

**Keywords:** miRNAs, Echinococcus, Immunomodulation, Host–parasite interaction, Diagnosis, Therapeutic strategies

## Abstract

**Background:**

MicroRNAs (miRNAs) are a subclass of small non-coding RNAs that regulate gene expression and play important roles in infectious diseases. Echinococcosis is a globally distributed disease caused by various species of *Echinococcus*. The disease can be asymptomatic in the early stages, but if left untreated, it can lead to severe complications. Like other helminths, *Echinococcus* spp. exhibit immunomodulatory capacities that favour infection establishment and progression. Currently, there is no fully accurate, non-invasive method for early detection of echinococcosis. This systematic review provides a comprehensive analysis of the existing literature and discusses recent advances in the study of miRNAs during host–*Echinococcus* spp. interactions, emphasizing their intriguing role in immunomodulation and their potential as biomarkers for detection, disease activity, prognosis, and therapeutic monitoring, as well as their possible relevance for future therapeutic research.

**Methods:**

This systematic review was conducted in accordance with the PRISMA 2020 guidelines. Searches were performed in PubMed, Web of Science, and Scopus without date restrictions to identify studies assessing the diagnostic potential of miRNAs in cystic echinococcosis (CE) and alveolar echinococcosis (AE). Fourteen original research articles were included in the final analysis.

**Results:**

Parasite-derived miRNAs have been detected in plasma, serum, and liver tissue, with egr-let-7-5p and egr-miR-71-5p emerging as candidates for CE detection and monitoring. In CE, dysregulation of host miRNAs was observed, displaying cyst activity-dependent patterns associated with inflammation, immunomodulation, and fibrosis, including a consistent upregulation of hsa-miR-146a-5p in plasma and serum samples. In AE, several host miRNAs exhibited altered expression in liver tissue and plasma, with hsa-miR-483-3p consistently upregulated. Comparative analyses revealed partially distinct but overlapping miRNA profiles in CE and AE, reflecting host-driven regulatory responses related to immunomodulation and tissue remodeling with stage-dependent patterns. Most included studies were based on case–control designs, with small sample sizes and limited validation, suggesting high overall risk of bias.

**Conclusions:**

Current evidence suggests that miRNAs have translational potential as complementary biomarkers for diagnosis, patient stratification, prognostic assessment, and disease monitoring in echinococcosis and may support future therapeutic research. Nonetheless, methodological heterogeneity and limited validation may contribute to overestimating diagnostic performance and hinder clinical translation.

**Graphical Abstract:**

**Figure:**
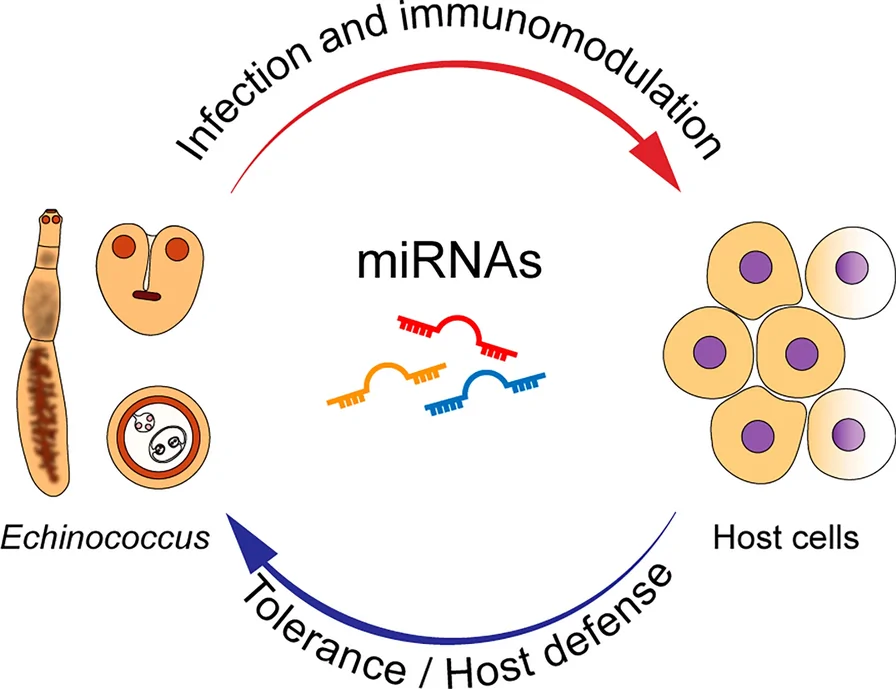

## Background

Echinococcosis ranks among the most severe and lethal zoonotic diseases affecting humans. Several cestode species from the genus *Echinococcus* (family Taeniidae) cause infection through their larval stages (metacestodes). Primarily, *Echinococcus granulosus* (*E. granulosus;* currently called *E. granulosus *sensu lato) and *Echinococcus multilocularis* (*E. multilocularis*) are responsible for cystic (CE) and alveolar echinococcosis (AE), respectively, the two most prevalent and clinically relevant forms of the disease [1, 2].

**Fig. 1:**
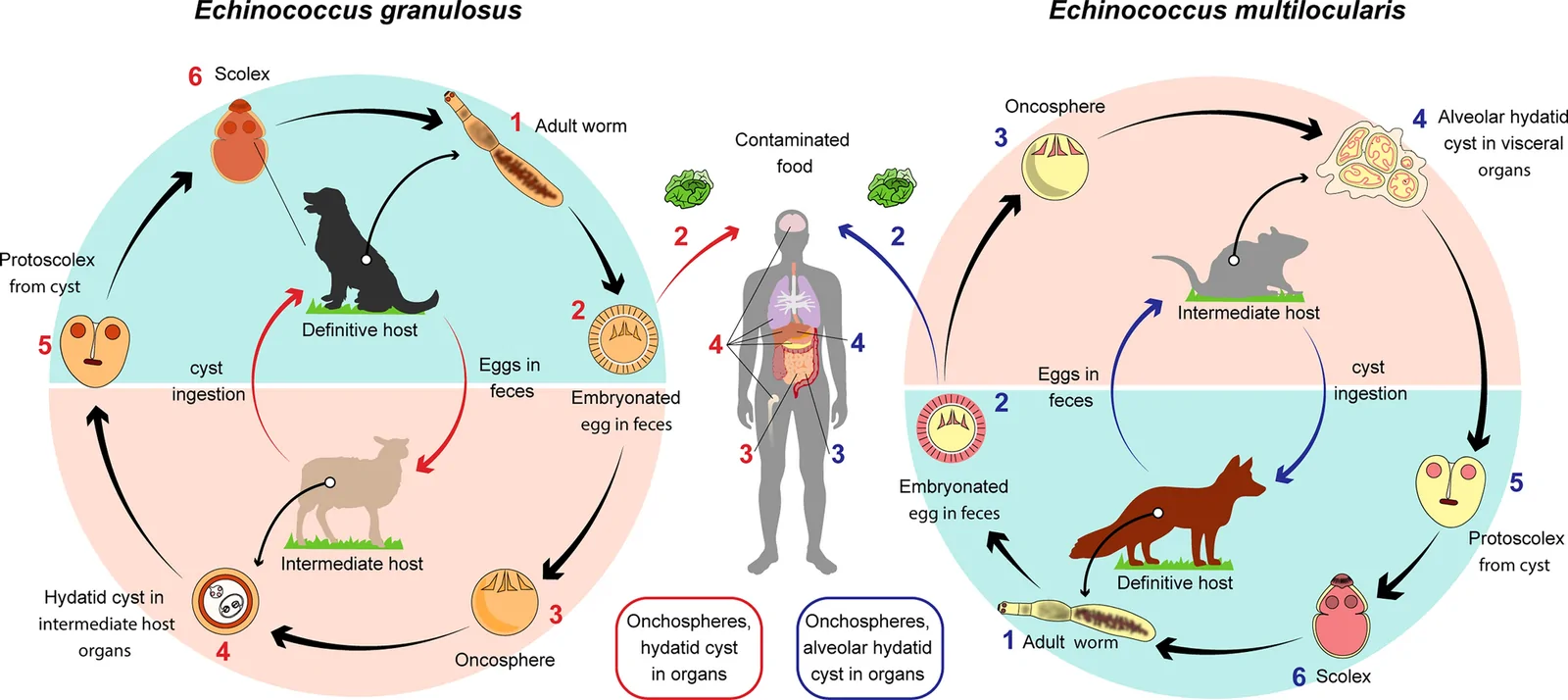
Life cycles of *Echinococcus* species.** A ***Echinococcus granulosus*. The adult parasite resides in the small intestine of definitive hosts (dogs or other canids). Following sexual maturation, gravid proglottids release eggs that are shed in feces. After ingestion by intermediate hosts (e.g., sheep, swine, goats, horses, cattle, camels), the eggs hatch, releasing oncospheres that penetrate the intestinal wall and migrate to organs, primarily the liver and lungs, where they form hydatid cysts. These cysts grow, producing protoscoleces (PSCs) and daughter cysts. The definitive host becomes reinfected by consuming organs containing viable cysts. PSCs then develop into adults, completing the cycle. Humans, as aberrant hosts, acquire infection via ingestion of eggs from contaminated food, water, or direct contact. Oncospheres migrate and form hydatid cysts in various organs. Cyst rupture can lead to secondary echinococcosis. **B ***Echinococcus multilocularis*. The cycle is similar but involves foxes and other carnivores as definitive hosts, and rodents as intermediate hosts. Ingested eggs hatch in the intestine, and oncospheres migrate primarily to the liver, where they form multilocular (alveolar) cysts characterized by exogenous budding and invasive growth. Humans become accidental intermediate hosts, developing hepatic cysts with potential for extrahepatic dissemination. The schematic illustration was created using Adobe Illustrator

Both species share a complex life cycle involving definitive carnivore hosts and intermediate mammalian hosts, with humans acting as accidental hosts. The developmental cycle comprises four stages: eggs, oncospheres, protoscoleces (PSCs), and adult forms (Figure 1). Adult parasites reside in the small intestine of the definitive host, where they release eggs into the environment [3, 4]. Following ingestion by intermediate hosts, *E. granulosus s.l.* larvae migrate mainly to target organs—predominantly the liver and lungs—where they develop into hydatid cysts filled with infectious PSCs, completing the life cycle upon ingestion by the definitive host (Figure 1A) [3, 5]. *E. multilocularis* follows a similar transmission route but is characterized by the formation of infiltrative multilocular cysts in intermediate hosts and humans, primarily affecting the liver and exhibiting invasive growth, mimicking metastatic tumors (Figure 1B) [4, 5]. These cystic stages are characterized by prolonged persistence, and the host–parasite interface they establish implies the existence of finely tuned regulatory mechanisms that sustain parasite survival, modulate host immunity, and enable long-term tissue adaptation [6].

Both *E. granulosus s.l*. and *E. multilocularis* exhibit substantial genetic diversity. The *E. granulosus s.l.* complex comprises several species that were historically classified as genotypes G1–G10. According to current consensus, these genotypes correspond to distinct species, including *E. granulosus sensu stricto* (G1–G3), *E. equinus* (G4), *E. ortleppi* (G5), and *E. canadensis* (G6–G10) [7]. In *E. multilocularis*, researchers have identified 29 genotypes and 26 haplotypes (A1–A26) [8–10]. These genotypic variations influence life cycle patterns, pathogenicity, drug susceptibility, and host and tissue tropism [11].

Recognizing its public health impact, the World Health Organization (WHO) included echinococcosis among the 21 neglected tropical diseases in 2021, with approximately one million people affected worldwide [12]. CE occurs globally, with endemic regions in Central Asia, Africa, South America, Australia, and the Mediterranean basin, whereas AE is mainly restricted to the Northern Hemisphere, particularly in China, Russia, parts of Europe, and North America [13]. Although AE has a lower incidence than CE, it is considerably more severe, with untreated mortality rates approaching 90%, compared with up to 40% for CE in the absence of appropriate healthcare [1]. According to the WHO, the lack of robust surveillance data, especially in remote areas and vulnerable populations, hinders the implementation and evaluation of effective control programs [12]. These factors highlight the need for diagnostic approaches that are adaptable to rural and vulnerable settings and that can enhance disease monitoring at both the population and clinical levels.

The diagnosis of echinococcosis is typically made several years after infection, usually following the onset of clinical symptoms [14]. Diagnosis relies mainly on imaging techniques combined with serological tests [1], while treatment options include surgery, percutaneous interventions, and prolonged benzimidazole (BZ) derivative-based chemotherapy, sometimes combined with other anthelmintic agents, depending on disease stage and clinical context [15]. However, this therapy is largely parasitostatic and frequently associated with adverse side effects [16]. Moreover, the performance of both diagnostic and therapeutic strategies demonstrates variable efficacy depending on disease stage and parasite species [17], limiting the timely diagnosis, accurate assessment of disease activity, and objective evaluation of treatment response. Overall, these limitations underscore the need for novel biomarkers and more targeted approaches for diagnosis, disease monitoring, and potentially for guiding therapeutic strategies.

In this context, increasing evidence suggests that non-coding RNAs, particularly microRNAs (miRNAs), play a key role in the pathogenesis of parasitic diseases, including echinococcosis [18–21]. MiRNAs are widely conserved molecules that regulate gene expression at the post-transcriptional level by binding to the 3' untranslated regions (3'UTRs) of target mRNAs, typically leading to translational repression or mRNA degradation [18, 19]. Through this regulatory capacity, miRNAs participate in diverse biological processes, including signal transduction, apoptosis, and immune response. Altered miRNA expression patterns have been shown to influence host–pathogen interactions, especially through their influence on immunomodulation [18, 19]. Given their regulatory capacity and remarkable stability in biological fluids, miRNAs have emerged as potential candidates for diagnostic and prognostic biomarkers [22], as well as therapeutic tools [18, 19].

Despite growing evidence on the role of miRNAs in host-parasite interactions, knowledge is limited, particularly in the context of human echinococcosis. Most available studies are experimental or focus on individual microRNAs, and there is a lack of comprehensive synthesis of microRNA profiles in patient studies, as well as of their potential applicability as biomarkers in clinical settings.

Considering these gaps and the limitations of existing diagnostic and therapeutic strategies, this systematic review aims to comprehensively synthesize current evidence on host- and parasite-derived miRNAs involved in *Echinococcus* spp. infections, with a particular emphasis on findings derived from patient-based studies. Additionally, it discusses their translational potential as diagnostic biomarkers for disease detection and activity assessment, prognostic evaluation, monitoring therapeutic response, and as candidate molecular targets for future therapeutic exploitation, along with the current limitations and challenges that hinder their clinical implementation.

To our knowledge, this represents the first systematic review specifically focused on miRNA research in human echinococcosis, integrating evidence from patient-based studies and encompassing both CE and AE forms of the disease.

### Role of miRNAs in helminthic infections

In helminthic infections, miRNAs act as key regulators of parasite development, immunomodulation, and stage-specific adaptation, contributing to immune evasion and survival within the host and, in some contexts, to drug resistance mechanisms [19, 23–26]. These effects are partly mediated by the release of parasite-derived miRNAs via extracellular vesicles (miRNA-EVs), which can be internalized by host cells and modulate genes involved in immune responses, metabolism, coagulation, and signal transduction [18, 19, 27]. In parallel, helminth infections have been shown to induce the differential expression of host-derived miRNAs that target inflammatory pathways [19, 28, 29]. Collectively, these findings suggest that miRNA-mediated immunomodulation represents a conserved strategy among helminths that may contribute to the establishment and maintenance of chronic infection and immune tolerance [19]. In this context, miRNAs have been proposed as potential targets to disrupt immune evasion mechanisms and improve parasite clearance [19, 30].

Similarly, *Echinococcus* spp. establish chronic infections associated with long-term immune tolerance; however, these infections are distinctly characterized by persistent hydatid cysts, which sustain prolonged and localized host–immune interactions within a relatively stable tissue microenvironment that can persist for years or even decades. In *Echinococcus* spp., miRNAs constitute one of the most abundant classes of small RNAs, accounting for approximately 5% to 69% of the total small RNA content, suggesting their potential relevance for miRNA-mediated regulation during infection [31–34]. Moreover, host miRNAs that are differentially expressed during *Echinococcus* spp. infections are broadly associated with fibrosis, angiogenesis, and immune tolerance; these processes are tightly linked to cyst dynamics and progressive parasite growth, thereby reflecting disease progression and chronic pathogenic features that distinguish CE and AE from other helminth infections [35, 36]. In contrast, in infections caused by intravascular helminths, such as *Schistosoma* spp.*,* circulating miRNAs have been proposed primarily as biomarkers of active infection or tissue damage rather than indicators of long-term disease dynamics [37–39].

**Fig. 2:**
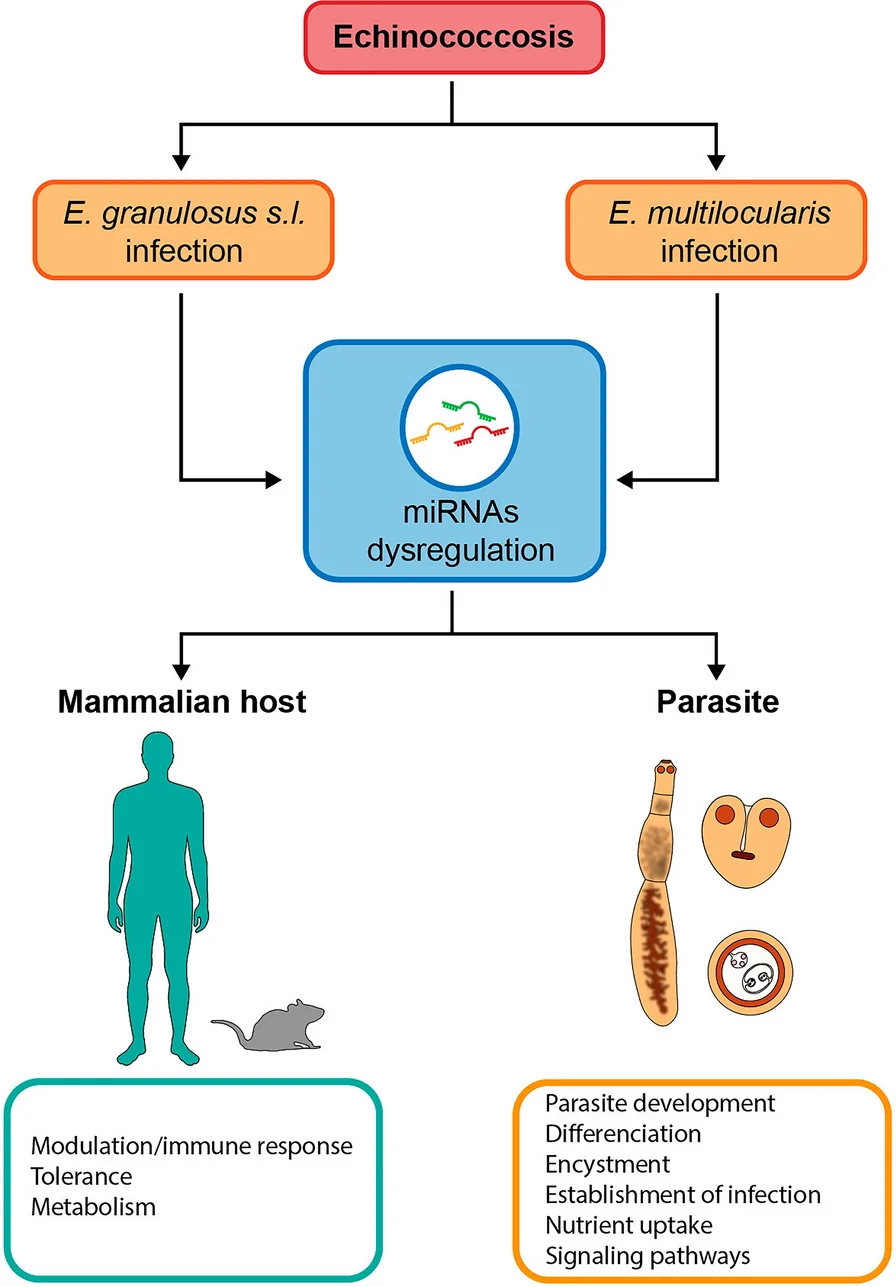
miRNAs during *Echinococcus* spp. pathogenesis. During *Echinococcus* spp. infection, miRNA expression is regulated in both the parasite (*E. granulosus s.l.* and *E. multilocularis*) and the host. Parasite-derived miRNAs are involved in the regulation of key biological processes, including development, differentiation, encystment, nutrient acquisition, and intracellular signaling pathways essential for parasite survival. In parallel, host-derived miRNAs are modulated in response to infection and contribute to the regulation of immune responses, immune tolerance, metabolic pathways, and tissue remodeling. Collectively, these miRNA-mediated mechanisms illustrate the dynamic molecular crosstalk underlying host–parasite interactions and their potential role in regulating immune responses, host metabolism, and parasite development during *Echinococcus* spp. infection. This figure was created using Adobe Illustrator

In *Echinococcus* spp., the temporal stability of cysts and the extreme chronicity of CE and AE suggest that miRNAs may have additional potential as biomarkers of cystic activity, disease progression, response to treatment, as well as candidates for future therapeutic research and molecular targets [36, 40]. For all the above reasons, *Echinococcus* spp. represents a particularly relevant model for the translational exploration of miRNA-based biomarkers and therapeutic strategies. In this context, the available evidence indicates that both parasite- and host-derived miRNAs act as key regulators of the parasite–host interface, influencing parasite development and host metabolism and immune responses during infection (Figure 2).

## Methods

### Literature search strategy

**Fig. 3:**
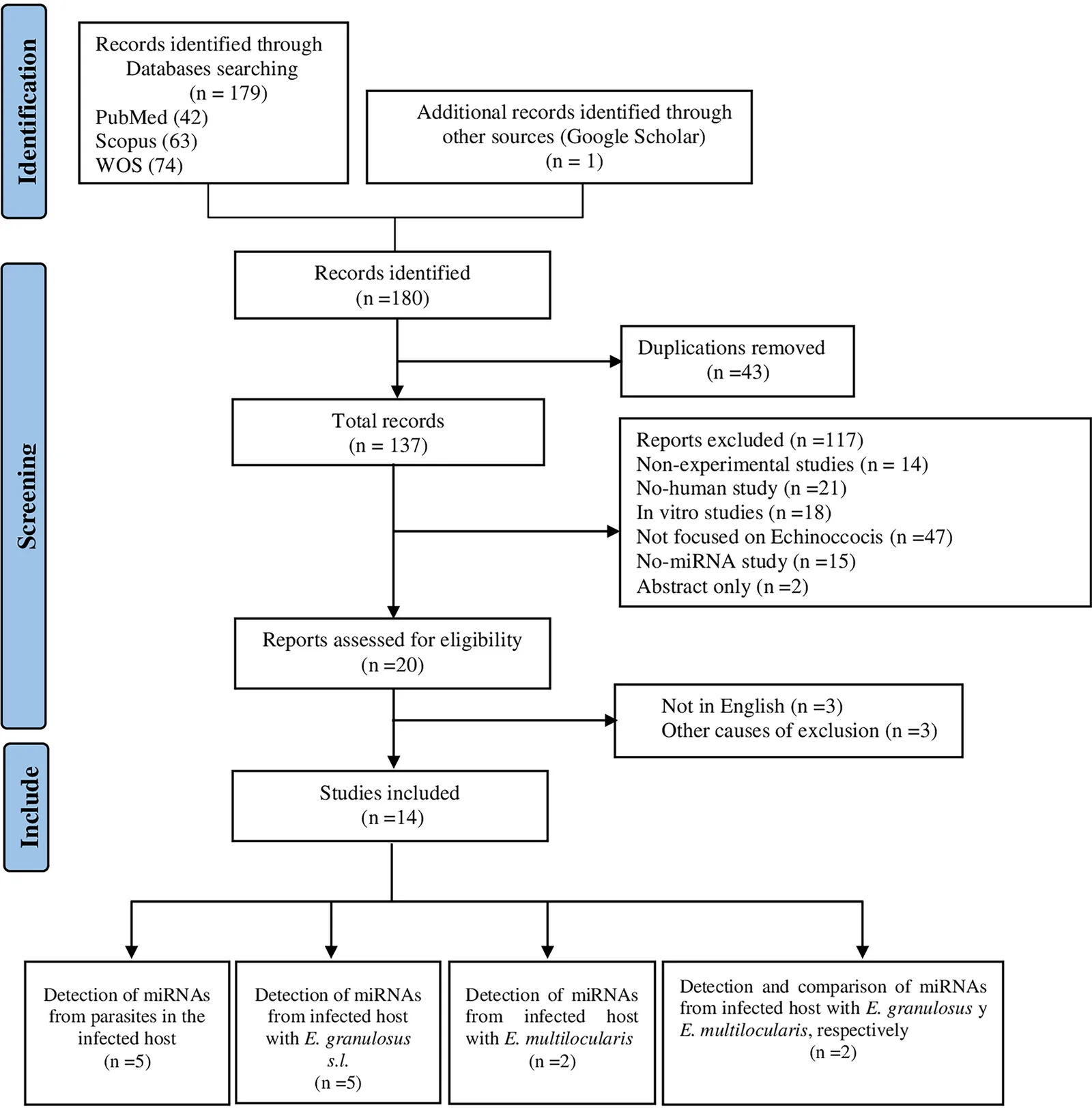
Flowchart of articles retrieved from the search of databases

To explore the role of miRNAs in *Echinococcus* spp. infections, we conducted a systematic review of studies assessing their diagnostic potential and therapeutic applications in cystic echinococcosis (CE) and alveolar echinococcosis (AE). This review was conducted and reported in accordance with the PRISMA 2020 guidelines [41]. The completed PRISMA checklist is provided in Table S1, and the study selection process is illustrated in the PRISMA flow diagram (Fig. 3).

We systematically searched PubMed, Web of Science (WOS), and Scopus from database inception to December 15, 2024. The following Boolean search strategy was used: (“MicroRNA” OR “microRNAs” OR “miRNA” OR “miRNAs” OR “miR”) AND (“*Echinococcus granulosus*” OR “hydatidosis” OR “cystic echinococcosis” OR “*E. granulosus*” OR “*Echinococcus multilocularis*” OR “alveolar echinococcosis” OR “*echinococcosis multilocularis*” OR “*E. multilocularis*”) AND (“Biomarkers” OR “Gene expression” OR “Molecular diagnosis”). No restrictions on study design were applied during the search.

Additionally, we conducted a manual search in Google Scholar to identify potentially relevant studies not indexed in the above databases. All retrieved records were imported into the Rayyan platform (https://www.rayyan.ai/) for duplicate removal and screening. We registered the review protocol under PROSPERO (CRD42024616237) before study selection. Two investigators (M.R.P. and D.A.A.) independently screened titles and abstracts for eligibility. Full-text articles of potentially relevant studies were subsequently assessed independently by the same reviewers. Any disagreements were resolved through discussion and consensus with senior authors (M.M. and U.K.).

### Article selection and data extraction

Studies were included if they met the following criteria: (i) original research articles, (ii) written in English, (iii) investigated human infections caused by *Echinococcus* spp., (iv) analyzed miRNAs using molecular techniques, and (v) used human-derived samples (e.g., blood, plasma, serum, exosomes, or liver tissue). Studies were required to evaluate the role of miRNAs as potential diagnostic biomarkers or to report expression profiles with potential diagnostic relevance (Table S2).

Non-experimental studies (e.g., reviews, editorials, commentaries, letters, conference abstracts) and articles focused on other parasites were excluded. Studies that did not involve microRNAs or use molecular approaches for their analysis, as well as those based exclusively on animal models or in vitro systems without human samples, were excluded. Articles for which the full text was unavailable were also excluded.

Data extraction was performed independently by two reviewers. The following information was collected from each eligible study: authorship, year of publication, sample type, analytical methodology, disease model, miRNAs investigated, their direction of regulation (up- or down-regulated), statistical significance (p < 0.05, based on each study’s analytical approach), and reported miRNA targets. The primary outcome was the identification of miRNAs with potential diagnostic relevance in human echinococcosis, defined as those showing statistically significant differential expression between infected and control groups or across disease stages, when reported. Secondary outcomes included their association with biological processes, disease stage, and potential clinical applications.

The studies were grouped into two categories: (I) those identifying *Echinococcus*-derived miRNAs in infected patients and (II) those profiling host miRNAs differentially expressed in response to *E. granulosus s.l*. or *E. multilocularis* infection.

For the purpose of data synthesis, the extracted variables were subsequently reorganized into predefined analytical categories to enable structured comparison across studies, including miRNA identity, biological origin (host or parasite), associated biological processes or pathways, and reported clinical relevance. Methodological quality was assessed using the Quality Assessment of Diagnostic Accuracy Studies (QUADAS-2) tool [42, 43], adapted to the exploratory and heterogeneous nature of the included miRNA expression studies by considering sample characterization, analytical methodology, and validation approaches. Since no validated risk-of-bias tool specifically exists for exploratory miRNA expression studies of human echinococcosis, the original QUADAS-2 domains were applied to evaluate methodological features potentially affecting biomarker validity and translational applicability rather than diagnostic accuracy itself. Because several included studies focused on differential expression or mechanistic aspects rather than formal diagnostic performance, the assessment should be interpreted as a structured evaluation of study quality within a translational context. The risk of bias was assessed across four domains (patient selection, index test, reference standard, and flow and timing), and applicability concerns were evaluated for patient selection, index test, and reference standard. All items were categorized as low, unclear, or high risk of bias.

Due to the heterogeneity of the included studies, no uniform effect measures could be defined across studies. Most studies reported relative miRNA expression levels (up- or down-regulation) and fold changes, together with statistical analyses based on each study’s predefined methodology. In some cases, diagnostic performance metrics such as sensitivity and specificity derived from ROC analyses were also reported. These measures were used descriptively according to the type of outcome reported in each study, given the heterogeneity of study designs and reported endpoints.

Although risk of bias within individual studies was assessed using QUADAS-2, a formal assessment of reporting bias was not feasible because of the limited number and variability of the included studies. However, the predominance of small, exploratory studies suggests a potential risk of publication bias.

In light of the substantial variability in study design, analytical platforms, and reported outcomes, a formal assessment of certainty of evidence using frameworks such as GRADE was not considered appropriate. Instead, the overall strength of the evidence was qualitatively evaluated based on study design, sample size, methodological consistency, and the presence or absence of validation cohorts, indicating a generally low to unclear level of evidence.

### Results and literature review

Our initial search identified 180 articles. After removing 43 duplicates, we screened the remaining 137 for relevance and eligibility. We excluded 121 articles based on title and abstract review. Ultimately, we included 14 original research articles in this systematic review (Fig. 3).

### Detection of parasite-derived miRNAs in human samples

A limited number of studies have reported detecting *Echinococcus*-derived miRNAs in human biological samples, including plasma, serum, and liver tissue. These miRNAs have demonstrated potential as biomarkers for diagnosis, disease staging, and monitoring of echinococcosis (Table 1).

**Table 1:** miRNAs of *E. granulosus s.l* and *E. multilocularis* detected in samples from patients with Echinococcosis

Origin	Sample	Detected miRNAs	Target pathways/biological processes	Clinical application	Method	References
*E. multilocularis*						
Parasite	Plasma	emu-miR-novel 1-3p	Unknown role in infection	Diagnostic biomarker of AE	miRNAseq/RT-PCR	Ma et al., 2024 [47]
*E. granulosus s.l*						
Parasite	Plasma	egr-let-7-5p egr-miR-71-5p	Host-parasite interaction, and parasite biology	Early diagnosis and monitoring of CE	RT-PCR	Alizadeh et al., 2020 [45]
Parasite	Serum	egr-let-7-5p egr-miR-71-5p egr-miR-9-5p	Regulation of the life cycle stages of the parasite	Stage-specific diagnosis	RT-PCR	Örsten et al., 2022 [46]
Parasite	Serum	egr-miR-2a-3p* egr-miR-125-5p¨* egr-miR-2a-3p #	Inflammation, collagen catabolism, and apoptosis	Diagnosing CE and monitoring its potential recurrence after cystectomy	RT-PCR	Karami et al., 2023 [16]
Parasite	Liver	egr-let-7-5p egr-miR-71-5p egr-miR-2a-3p egr-miR-125-5p	Modulation of inflammatory response, parasite-host interactions, collagen catabolism, apoptosis, and the mitogen-activated protein kinase (MAPK) cascade	Early detection of CE Predicting liver fibrosis	RT-PCR	Karami et al., 2023 [48]

^*^Detected in active CE, # Detected after the surgery

In patients with AE, one study reported the detection of emu-miR-novel-1-3p in plasma samples, suggesting its utility in AE diagnosis [44].

In CE, several studies consistently detected egr-let-7-5p and egr-miR-71-5p in plasma and serum samples, in association with hydatid cyst pathogenesis and parasite–host interaction [16, 45, 46]. Additional serum sample-based studies identified egr-miR-2a-3p and egr-miR-125-5p, which have been reported to be associated with inflammatory processes, collagen catabolism, apoptosis, and host–parasite interactions. In these studies, miRNAs were proposed as biomarkers for CE diagnosis and for monitoring potential disease recurrence following cystectomy [16].

In liver tissue, studies revealed that samples from patients with CE showed the presence of several *E. granulosus s.l* miRNAs, including egr-let-7-5p, egr-miR-71-5p, egr-miR-2a-3p, and egr-miR-125-5p. These miRNAs were associated with pathways related to inflammatory responses, parasite–host interactions, collagen catabolism, apoptosis, and activation of the mitogen-activated protein kinase (MAPK) signaling cascade.

Across studies, parasite-derived miRNAs such as egr-let-7-5p and egr-miR-71-5p were consistently detected in circulation and liver tissue, suggesting a potential role as diagnostic and disease-monitoring biomarkers in CE.

### Host-derived miRNA expression in *E. granulosus s.l.* infections

Table 2 summarizes the studies reporting host-derived miRNA dysregulation in patients infected with *E. granulosus s.l*. These studies analyzed different biological matrices, including serum, plasma, plasma-derived exosomes, and liver tissue, and reported miRNA expression patterns that varied according to disease stage, particularly between active and inactive CE.

**Table 2:** Deregulated miRNAs in patients infected with *E. granulosus s.l*

Origin	Sample	Significant miRNAs	Regulation	Disease stage	Target pathways/biological processes	Clinical application	Method	Reference
Host	Serum	hsa-miR-146a-5p	Upregulated	AC	Inflammation, immune regulation	Diagnostic biomarker	RT-PCR	Mahmoud et al., 2024 [49]
Host	Plasma	hsa-miR-146a-5p hsa-miR-155	Upregulated	AC	Immune response, Th2 regulation	Diagnostic biomarker and severity of hydatidosis	RT-PCR	Mahami-Oskouei et al., 2021 [50]
Host	Exosomes from the plasma	hsa-let-7a-3p hsa-let-7i-3p hsa-let-7i-5p hsa-miR-15a-3p hsa-miR-15b-5p hsa-miR-16–1-3p hsa-miR-17-3p hsa-miR-18b-5p hsa-miR-20a-3p hsa-miR-29c-3p hsa-miR-93-5p hsa-miR-103a-3p hsa-miR-107 hsa-miR-103a-3p hsa-miR-140-3p hsa-miR-140-5p hsa-miR-210-3p hsa-miR-132-3p hsa-miR-142-3p hsa-miR-335-5p hsa-miR-484 hsa-miR-548e-3p hsa-miR-556-3p hsa-miR-7976_R + 1 hsa-let-7a-5p hsa-let-7f-5p hsa-let-7e-5p hsa-miR-146a-5p hsa-miR-146b-5p hsa-miR-30a-5p hsa-miR-30b-5p hsa-miR-30c-5p hsa-miR-30d-5p hsa-miR-30e-5p hsa-miR-320c hsa-miR-320d hsa-miR-199a-3p hsa-miR-99b-5p hsa-miR-155-5p hsa-miR-214-3p hsa-miR-223-5p hsa-miR-125b-5p hsa-miR-195-5p hsa-miR-145-5p hsa-miR-338-5p	Upregulated Downregulated	AC	Immune modulation, metabolism, tissue remodulation and host–parasite interaction	Therapeutic target Prediction of the mechanism of the host-parasite interactions	Deep sequencing	Xiao et al., 2024 [51]
Host	Serum	hsa-miR-4659a-5p hsa-miR-4518 hsa-miR-3977 hsa-miR-4692 hsa-miR-181b-3p hsa-miR-4491 hsa-miR-4687-5p	Downregulated Downregulated	AC IC	Apoptosis, proliferation, cell–cell interactions, and cell cycle	Diagnostic biomarker Key molecules to study the relation between cancer development and CE	miRNA PCR Array	Orsten et al., 2021 [36]
Host	Liver	hsa-miR-19b-3p	Downregulated	AC	TGF-β signaling, fibrogenesis	Therapeutic target	RT-PCR	Zhang et al., 2016 [52]

^*^ *AC* Active, *IC* inactive cysts

In serum and plasma samples from patients with active CE, hsa-miR-146a-5p was consistently reported as upregulated and was associated with inflammation and immune regulation [49–51]. In these studies, this miRNA was proposed as a potential biomarker for disease presence and for assessing disease severity.

Analysis of plasma-derived exosomes revealed a broader panel of deregulated host miRNAs in patients with CE [51]. Upregulated miRNAs included members of several conserved miRNA families, such as the let-7 family (hsa-let-7a-3p, hsa-let-7i-3p, hsa-let-7i-5p), the miR-15/16 family (hsa-miR-15a-3p, hsa-miR-15b-5p, hsa-miR-16-1-3p), and the miR-17~92 cluster (hsa-miR-17-3p, hsa-miR-18b-5p, hsa-miR-20a-3p). Additional upregulation was reported for the miR-29 family (hsa-miR-29a-3p, hsa-miR-29b-3p, hsa-miR-29c-3p), the miR-103/107 family (hsa-miR-103a-3p, hsa-miR-107), the miR-140 family (hsa-miR-140-3p, hsa-miR-140-5p), and the miR-181 family (hsa-miR-181a-2-3p). Other miRNAs, including hsa-miR-132-3p, hsa-miR-93-5p, hsa-miR-335-5p, hsa-miR-101-3p, and hsa-miR-148b-3p, which were also reported as upregulated in patients with active CE [51].

In contrast, a subset of host-derived miRNAs was reported to be downregulated, including members of the let-7 family (hsa-let-7a-5p, hsa-let-7f-5p, hsa-let-7e-5p), the miR-146 family (hsa-miR-146a-5p and hsa-miR-146b-5p), the miR-30 family (hsa-miR-30a-5p, hsa-miR-30b-5p, hsa-miR-30c-5p, hsa-miR-30d-5p, and hsa-miR-30e-5p), the miR-320 family (hsa-miR-320c and hsa-miR-320d), and the miR-99 family (hsa-miR-199a-3p and hsa-miR-99b-5p). Additional downregulated miRNAs included hsa-miR-155-5p, hsa-miR-214-3p, hsa-miR-223-5p, hsa-miR-125b-5p, hsa-miR-195-5p, hsa-miR-145-5p and hsa-miR-338-5p.

Interestingly, some miRNAs, such as hsa-miR-146a-5p, showed opposite regulation patterns depending on the biological compartment analyzed, suggesting compartment-specific miRNA dynamics during CE.

In these studies, the downregulated miRNAs were reported to be associated with various biological processes, including immune modulation, fibrosis, angiogenesis, liver pathology, and the resolution of inflammation. In addition, these miRNAs were suggested as potential biomarkers and as molecules that may be involved in host–parasite interaction mechanisms [51].

In studies examining the relationship between miRNA expression and liver fibrosis, a decrease in hsa-miR-19b-3p expression was observed in the hepatic tissue of CE patients and was reported to be associated with enhanced TGF-β signaling and fibrogenesis. Based on these observations, the downregulation of hsa-miR-19b-3p may be associated with the development of pericystic fibrosis, and this miRNA has been suggested as a potential therapeutic target to modulate fibrosis-associated pathways [52].

In addition, only one serum-based study, including patients with both active and inactive cysts, evaluated host-derived miRNA expression according to cyst activity status. In this study, six miRNAs (hsa-miR-4659a-5p, hsa-miR-4518, hsa-miR-3977, hsa-miR-4692, hsa-miR-181b-3p, hsa-miR-4491) were downregulated in CE patients with active cysts, whereas one miRNA (hsa-miR-4687-5p) was downregulated in patients with inactive cysts, compared with control individuals. This study suggested that these miRNAs are associated with cellular processes such as apoptosis, proliferation, cell–cell interactions, and cell cycle regulation. Based on these associations, they have been proposed as candidate biomarkers and as molecular tools to explore potential links between CE-associated tissue remodeling and pathways commonly implicated in cancer-related processes [36].

Overall, host-derived miRNAs in CE were mainly reported to be associated with immunomodulation, fibrosis, and tissue remodeling, with several candidates suggesting potential roles in disease staging, activity assessment, and therapeutic targeting

### Host-derived miRNA expression in *E. multilocularis* infections

Studies included in Table 3 report host-derived miRNA dysregulation in patients with *E. multilocularis* infection, based on analyses of plasma and liver samples. Most of the reported miRNAs were associated with early-stage liver alveolar echinococcosis (LAE).

**Table 3:** Deregulated miRNAs in patients infected with *E. multilocularis*

Origin	Sample	Significant miRNAs	Regulation	Disease stage	Target pathways/biological processes	Application	Method	Reference
Host	Plasma	hsa-miR-483-3p	Upregulated	Early stage of LAE	Regulation of the immune response	Diagnostic biomarker of LAE	miRNA microarray/ RT-PCR	Ren et al., 2019 [53]
Host	Liver	hsa-miR-483-3p hsa-miR-1237-3p hsa-miR-33b-3p hsa-miR-4306	Upregulated Downregulated					
Host	Plasma and liver	hsa-miR-125b-5p	Upregulated	Not specified	Apoptosis regulation	Diagnostic biomarker of LAE	miRNA chips/ RT-PCR	Deping et al., 2021 [54]

Among patients with LAE, hsa-miR-1237-3p, hsa-miR-33b-3p, and hsa-miR-483-3p were reported to be upregulated in liver tissue, whereas hsa-miR-4306 was reported to be downregulated compared with controls. Notably, hsa-miR-483-3p was reported to be upregulated in both liver tissue and plasma samples, suggesting its potential as a diagnostic biomarker [53].

Another study reported that hsa-miR-125b-5p was upregulated in both plasma and liver tissue samples from AE patients. This miRNA has been associated with the regulation of apoptosis and was proposed as a potential diagnostic biomarker, although the disease stage of the patients was not specified in this study [54].

Collectively, host-derived miRNAs in AE were mainly reported to be associated with immune regulation, apoptosis, and liver pathology, with selected candidates suggesting a potential role for diagnostic applications, particularly in early-stage disease.

### Comparative miRNA expression in CE and AE

Table 4 presents two studies comparing miRNA expression between CE and AE patients. In these studies, deregulated host-derived miRNAs were analyzed in liver tissue and serum samples from CE and AE patients.

**Table 4:** Comparison of miRNAs deregulated in patients infected with *E. granulosus s.l.* and *E. multilocularis*

Origin	Sample/ tissue	Parasite causing the infection	Significant miRNAs	Regulation	Disease stage	Target pathways/biological processes	Clinical application	Method	Reference
Host	Liver	*E* *Granulosus s.l*	hsa-miR-15a-5p hsa-miR-21-5p hsa-miR-27a-3p hsa-miR-29a-3p hsa-miR-146a-5p hsa-miR-181a-5p hsa-miR-181b-5p hsa-miR-181d hsa-miR-181c-5p hsa-miR-195-5p hsa-miR-214-3p hsa-miR-365-3p	Upregulated	Initial CE	Regulation of immune tolerance and homeostasis of the host	Early diagnosis of Echinococcosis Therapeutic target	RT-PCR	Eroglu et al., 2021 [55]
		*E. multilocularis*	hsa-let-7a-5p hsa-let-7c hsa-let-7e-5p hsa-miR-15b-5p hsa-miR-16 hsa-miR-17-5p hsa-miR-23a-5p hsa-miR-24-3p hsa-miR-25-3p hsa-miR-26a-3p hsa-miR-26b-3p hsa-miR-29b-3p hsa-miR-29c-3p hsa-miR-30a-5p hsa-miR-30b-5p hsa-miR-30c-5p hsa-miR-30d-5p hsa-miR-30e-5p hsa-miR-98-5p	Downregulated	Initial or moderate AE				
			hsa-miR-101-3p hsa-miR-106b-5p miR-125b-5p miR-142-5p miR-222-3p miR-223-3p						
Host	Serum	*E. multilocularis*	AE patients positive hsa-miR-122-5p hsa-miR-1246 hsa-miR-144-5p AE treated hsa-miR-150-5p hsa-miR-122-5p hsa-miR-483-5p hsa-miR-144-5p hsa-miR-324-5p hsa-miR-485-3p hsa-miR-374a-5p	Upregulated Downregulated Upregulated Downregulated	Active (with liver lesion) Positive (treated with ABZ)	Modulation of immunomodulatory processes	Biomarkers for classifying patients into different categories Biomarkers for detection of helminths and differential diagnosis	sRNA-seq	Cucher et al., 2023 [56]
		*E* *granulosus s.l*	CE active hsa-miR-1246 hsa-miR-671-5p hsa-miR-423-5p hsa-miR-192-5p hsa-miR-125b-5p CE inactive hsa-miR-1246 hsa-miR-671-5p hsa-miR-423-5p hsa-miR-192-5p hsa-miR-1-3p CE treated hsa-miR-1246 hsa-miR-671-5p hsa-miR-423-5p hsa-miR-192-5p hsa-miR-125b-5p hsa-miR-125a-5p hsa-miR-590-3p NPL hsa-miR-1246 hsa-miR-671-5p hsa-miR-423-5p hsa-miR-125b-5p hsa-miR-431-5p	Upregulated Downregulated Upregulated Downregulated Upregulated Downregulated Upregulated Downregulated	In different stages of disease (CE1-2/ CE3a and CE3b) Stage CE4-CE5 Stage CE1-2 treated with ABZ				

Patient’s groups: CE inactive infection (CE inactive), CE active infection (CE positive), CE treated patients (CE treated), CE negative patients with non-parasitic lesion (NPL), albendazole (ABZ)

### Liver tissue

A comparative analysis of liver tissue from patients with CE and AE, with healthy controls, identified partially overlapping patterns of deregulated host miRNAs rather than completely disease-specific signatures. In patients with CE, several miRNAs were reported to be upregulated compared with control samples, including hsa-miR-15a-5p, hsa-miR-21-5p, hsa-miR-27a-3p, hsa-miR-29a-3p, hsa-miR-146a-5p, members of the hsa-miR-181 family (hsa-miR-181a-5p, hsa-miR-181b-5p, hsa-miR-181c-5p, and hsa-miR-181d), hsa-miR-195-5p, hsa-miR-214-3p, and hsa-miR-365-3p. In contrast, the downregulated panel in patients with early or unclear AE included members of the let-7 family (let-7a-5p, let-7c, let-7e-5p), the hsa-miR-15/16 cluster (hsa-miR-15b-5p, hsa-miR-16), hsa-miR-26 family (hsa-miR-26a-3p, hsa-miR-26b-3p), the hsa-miR-29 family (hsa-miR-29b-3p, hsa-miR-29c-3p), the hsa-miR-30 family (hsa-miR-30a-5p, hsa-miR-30b-5p, hsa-miR-30c-5p, hsa-miR-30d-5p, hsa-miR-30e-5p), hsa-miR-17-5p, hsa-miR-23a-5p, hsa-miR-24-3p, hsa-miR-25-3p, hsa-miR-98-5p, hsa-miR-101-3p, hsa-miR-106b-5p, hsa-miR-125b-5p, hsa-miR-142-5p, hsa-miR-222-3p, and hsa-miR-223-3p.

Although different family members and opposite regulation patterns were reported, both CE and AE involved deregulation of the miR-29 and miR-15/16 families, suggesting partially shared host regulatory responses rather than completely disease-specific liver signatures. These deregulated miRNAs were associated with immune tolerance and host immune homeostasis and have been proposed as candidates for early diagnosis and future therapeutic exploration in echinococcosis.

### Serum samples

Analyses performed on serum samples identified stage- and treatment-dependent miRNA expression patterns in patients with AE and CE when compared with control groups.

In patients with AE and active liver lesions, dysregulation of several miRNAs was reported, including hsa-miR-122-5p and hsa-miR-1246, which were upregulated, whereas hsa-miR-144-5p was downregulated relative to controls. In albendazole-treated AE patients, altered expression of other miRNAs was described. Specifically, hsa-miR-150-5p, hsa-miR-122-5p, and hsa-miR-483-5p were reported as upregulated, whereas hsa-miR-144-5p, hsa-miR-324-5p, hsa-miR-485-3p, and hsa-miR-374a-5p were downregulated. Notably, hsa-miR-122-5p was consistently upregulated, whereas hsa-miR-144-5p remained consistently downregulated across both active and treated AE groups.

In CE patients, serum miRNA profiles were reported to be largely conserved across cyst activity states, with differences restricted to a limited number of miRNAs. Accordingly, across all CE patient groups analyzed, including active CE, inactive CE (stages CE4–CE5), albendazole-treated CE (CE1-CE3 cysts), and non-parasitic liver lesions (NPLs), hsa-miR-1246, hsa-miR-671-5p, and hsa-miR-423-5p were reported to be consistently upregulated, whereas hsa-miR-192-5p and members of the miR-125 family (hsa-miR-125b-5p and hsa-miR-125a-5p) were reported to be consistently downregulated. Differences between groups were reported to be limited to a small subset of miRNAs. Inactive CE was characterized by the downregulation of hsa-miR-1-3p, treated CE showed selective downregulation of hsa-miR-590-3p, and NPLs were reported to be distinguished by the downregulation of hsa-miR-431-5p.

These miRNAs were reported to be associated with immunomodulatory processes and have been proposed as potential candidate biomarkers for patient stratification, detection of echinococcosis, and differential diagnosis.

Comparative analyses indicate that many miRNA alterations in CE and AE reflect shared host-driven regulatory pathways, particularly those related to immune tolerance and tissue remodeling, while distinct expression patterns may provide additional value for disease-specific profiling and clinical stratification, although these observations are based on a limited number of exploratory studies.

### Risk-of-bias assessment

When assessing the risk of bias in the studies included in this work, it was found that, overall, the risk of bias was predominantly high (Table 5). No study was considered to have a low risk of bias across all QUADAS-2 domains, while most studies showed high or unclear risk across multiple domains. The patient selection domain represented the main source of bias. Most studies employed case–control designs, typically comparing patients with confirmed echinococcosis to healthy controls. This design was consistently associated with a high risk of bias and spectrum bias due to the limited representativeness of the clinical populations, as it compares clearly diseased individuals with healthy controls rather than patients with clinically suspected disease. Only a small number of studies included more structured or clinically oriented sampling approaches. In addition, some studies evaluated differential miRNA expressions using intra-patient comparisons (e.g., pericystic tissue versus healthy liver tissue), while others included patients already selected for surgical intervention, which may introduce additional sources of selection bias.

**Table 5:** QUADAS-2 risk-of-bias assessment

	Risk of bias				Applicability concerns		
Study	Patient selection	Index test	Reference standard	Flow and timing	Patient selection	Index test	**Reference standard**
Ma et al. 2024 [47]	High	Unclear	Unclear	High	High	Moderate	Low
Alizadeh et al.,2020 [45]	High	Unclear	Low	Unclear	High	Unclear	Low
Örsten et al. 2022 [46]	High	Unclear	Low	Unclear	High	Unclear	Low
Karami et al. 2023 [16]	High	Unclear	Unclear	Unclear	High	Unclear	Unclear
Karami et al. 2023 [48]	High	Unclear	Low	Unclear	High	High	Low
Mahmoud et al. 2024 [49]	High	Unclear	Unclear	Unclear	High	Unclear	Low
Mahami-Oskouei et al. 2021 [50]	High	Unclear	Low	Unclear	High	Unclear	Low
Xiao et al. 2024 [51]	High	Unclear	Unclear	Unclear	High	High	Unclear
Orsten et al. 2021 [36]	High	Unclear	Low	Unclear	High	Unclear	Low
Zhang et al. 2016 [52]	High	High	Unclear	Unclear	High	High	Unclear
Ren et al. 2019 [53]	High	Unclear	Unclear	Unclear	High	Unclear	Low
Deping et al. 2021 [54]	High	Unclear	Unclear	Unclear	High	Unclear	Low
Eroglu et al. 2021 [55]	High	High	Low	Unclear	High	High	Low
Cucher et al. 2023 [56]	Unclear	Unclear	Low	Unclear	Unclear	High	Low

In the index test domain, methodological approaches were generally well described, and most studies used qRT-PCR or sequencing-based techniques. However, the formal assessment of diagnostic performance was reported inconsistently. Only a minority of studies included ROC analyses or provided measures such as sensitivity and specificity, while most relied on differential expression analyses without predefined thresholds or validation cohorts. As a result, this domain was frequently rated as unclear or at a high risk of bias. In studies reporting diagnostic performance, estimates of accuracy should be interpreted with caution, as methodological limitations—particularly case–control designs and lack of external validation—may lead to inflated sensitivity and specificity values. The reference standard domain was generally based on established clinical criteria, including imaging techniques, serological tests, and histopathological confirmation. Despite this, several studies lacked sufficient methodological detail regarding diagnostic procedures and blinding, leading this domain to be rated as unclear in those cases, although the reference standard was generally considered appropriate.

Similarly, the flow and timeline domains were insufficiently reported in most studies. Few studies provided detailed information on the timing of sample collection in relation to diagnosis, or confirmed that all included participants were accounted for in the final analysis. Furthermore, in several exploratory or tissue-based studies, assessment of this domain was limited by the absence of a clearly defined diagnostic pathway and was therefore rated as unclear.

Overall, only a small subset of studies evaluated miRNAs within a clinically relevant diagnostic framework, including validation cohorts and quantitative performance metrics. Furthermore, most studies were exploratory, focusing on miRNA profiling, differential expression, or mechanistic analyses. Additionally, a distinction should be made between parasite-derived and host-derived miRNAs. A substantial proportion of studies focused on host-response miRNAs, which may lack disease specificity, further limiting their applicability and generalizability as potential biomarkers. External validation in independent cohorts was largely absent across studies. Concerns regarding applicability were mainly associated with non-representative patient populations, exploratory study designs, and the use of host-derived miRNAs with limited disease specificity, potentially restricting the translation of these findings into routine clinical practice.

Taken together, the overall strength of the available evidence was considered low to unclear, with most evidence leaning toward low certainty due to small sample sizes, lack of validation cohorts, and methodological heterogeneity across studies.

## Discussion

### Detection of parasite-derived miRNAs in human samples

To date, only a limited number of studies have explored the diagnostic and translational potential of parasite-derived miRNAs in human echinococcosis. In patients with CE, parasite-derived miRNAs have been investigated under different clinical contexts, including detection, diagnosis, pathogenesis, and staging, and across sample types [16, 45, 46, 48]. Despite this heterogeneity, a subset of parasite-derived miRNAs, particularly egr-miR-71-5p and egr-let-7-5p, is recurrently identified across independent studies, supporting their potential relevance as biomarkers of infection and disease monitoring.

Changes in miRNA expression have been reported in relation to cyst removal and, in some studies, across different cyst stages. In addition, egr-miR-2a-3p has been identified in both circulating and tissue-based analyses within the same study framework, suggesting potential relevance across different biological contexts, although independent validation remains limited. These findings also indicate that parasite-derived miRNAs may be associated with fibrosis-related processes in hepatic tissue, reflecting local host–parasite interactions rather than circulating biomarker profiles. Although these results support the potential of parasite-derived miRNAs as candidate biomarkers for active infection, current studies are still limited by small sample sizes and the absence of independent validation cohorts, restricting immediate clinical applicability.

**Fig. 4:**
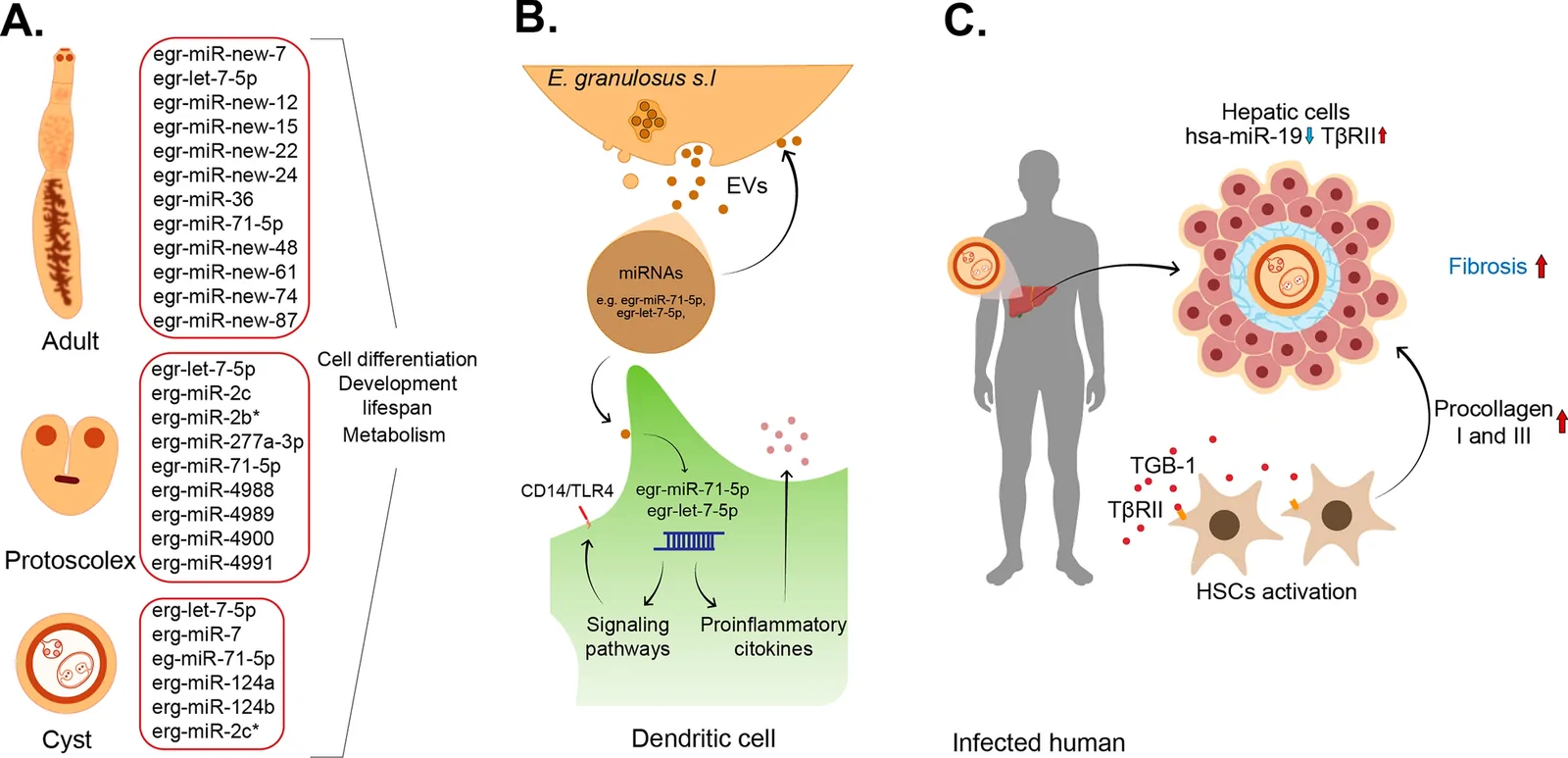
miRNAs in *E. granulosus s.l.* infection. During *E. granulosus s.l*. infection, both parasite- and host-derived miRNAs contribute to the regulation of key biological processes involved in parasite development and host response. **A** Stage-specific expression patterns of *E. granulosus s.l.* miRNAs across developmental stages, highlighting their involvement in parasite differentiation, development, metabolism, and lifespan regulation. **B** Parasite-derived miRNAs delivered via extracellular vesicles (EVs), including egr-miR-71-5p and egr-let-7-5p, may modulate host immune responses by regulating proinflammatory cytokine expression and key signaling pathways such as TLR4/IRF5/CD14. **C** In the host liver, *E. granulosus s.l.* infection has been associated with downregulation of hsa-miR-19, which promotes pericystic fibrosis through increased TGF-β receptor II (TβRII) expression, activation of hepatic stellate cells (HSCs), and enhanced extracellular matrix (ECM) deposition, including procollagen I and III, ultimately leading to liver fibrosis. Collectively, these processes illustrate the integrated role of parasite- and host-derived miRNAs in parasite survival, immune modulation, and host tissue remodeling. This illustration was created using Adobe Illustrator

The recurrent detection of egr-miR-71-5p and egr-let-7-5p in human samples is consistent with experimental data indicating that these miRNAs are among the most abundantly expressed across the parasite life cycle. Studies reported the identification of 114 mature miRNAs in *E. granulosus s.l*. Some miRNAs are constitutively expressed across all developmental stages, while others exhibit stage-specific expression patterns [31, 57, 58] (Figure 4A). These stage-dependent expression profiles suggest potential functional specialization; however, their direct relevance for biomarker development in human infection remains unclear.

Among these, egr-miR-71-5p and egr-let-7-5p are highly expressed throughout the parasite life cycle and have been reported to be most abundant in PSC and in the cyst wall, accounting for approximately 50% of total miRNA expression. This observation has led to the suggestion that these miRNAs may be essential for *E. granulosus s.l.* survival [33]. Although the specific functions of egr-miR-71-5p and egr-let-7-5p in this parasite remain unknown, they have been implicated in immune modulation and parasite pathogenesis [19, 59]. In addition, these miRNAs have been detected in hydatid fluid and in PSC-derived extracellular vesicles (PSC-EVs), which share seed sequences with host miRNAs. This raises the possibility that *E. granulosus s.l*. miRNAs may mimic host regulatory molecules to modulate host responses [60–62]. Experimental evidence indicates that these EV-delivered miRNAs can be internalized by host immune cells and alter cytokine expression (e.g., IL-1β, IL-10, IL-17, TNF-α) as well as modulate signaling pathways, such as TLR4/IRF5/CD14 [61] (Figure 4B). Most of these observations derive from experimental or in vitro models. Importantly, while these data support a role for parasite-derived miRNAs in host–parasite communication, it is not yet clear to what extent EV-associated miRNAs are consistently detectable in circulation or directly contribute to the miRNA signatures identified in human plasma or serum, highlighting a gap between mechanistic and translational evidence.

**Fig. 5:**
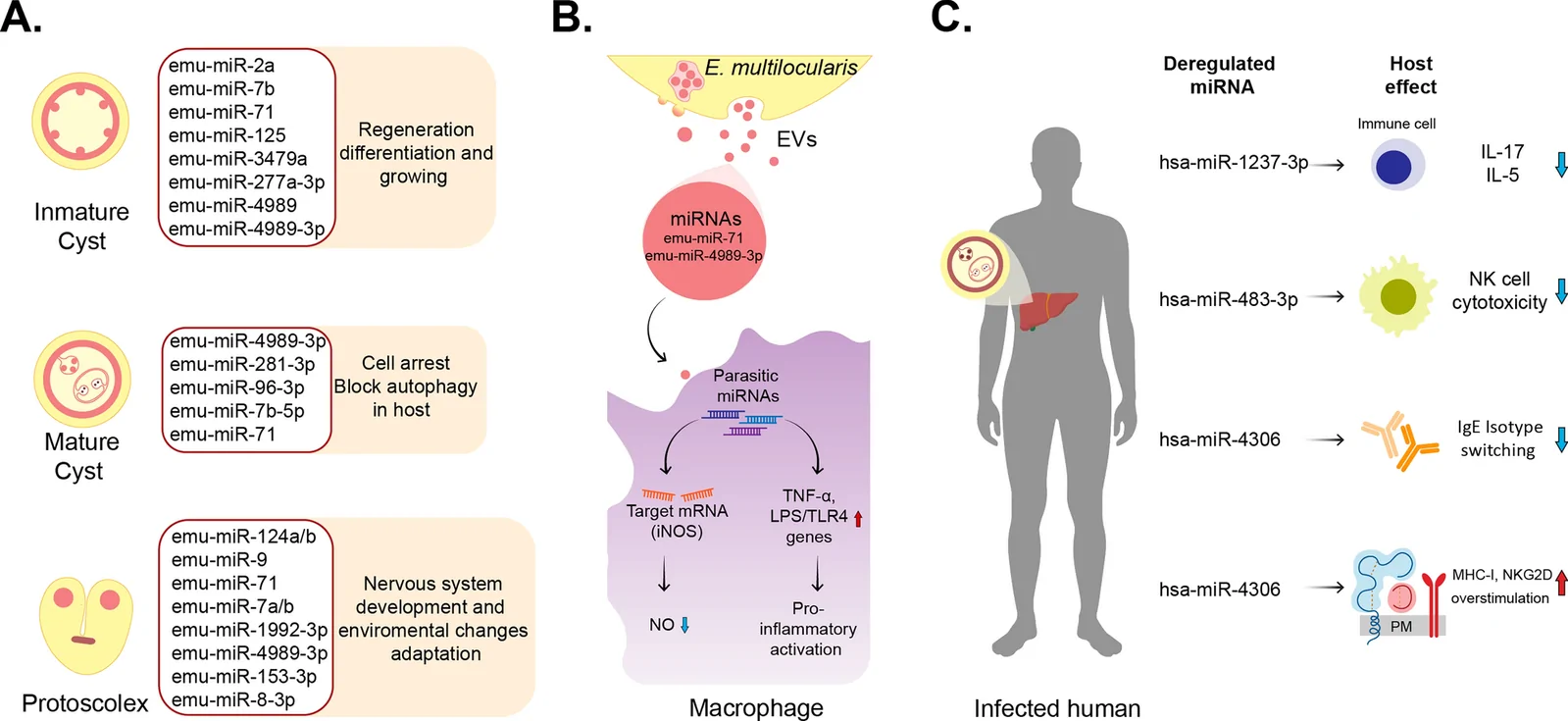
miRNAs in *E. multilocularis* infection. During infection, both parasite- and host-derived miRNAs contribute to the regulation of developmental processes and host immune responses. **A** Stage-specific expression patterns of *E. multilocularis* miRNAs across developmental stages, highlighting their involvement in regeneration, nervous system development, environmental adaptation, and host modulation. Some of these miRNAs (e.g., emu-miR-281-3p, emu-miR-96-3p, and emu-miR-7b-5p) have been associated with host cell cycle arrest and inhibition of autophagy. **B** Parasite-derived miRNAs delivered via extracellular vesicles (EVs), including emu-miR-71 and emu-miR-4989-3p, have been shown to modulate macrophage responses in experimental models by reducing nitric oxide (NO) production and influencing inflammatory signaling pathways such as TNF-α and LPS/TLR4, suggesting a modulatory capacity that may contribute to the pathogenesis of *E. multilocularis*. **C** In AE, altered expression of host-derived miRNAs, including hsa-miR-1237-3p, hsa-miR-483-3p, and hsa-miR-4306, is associated with the regulation of key immune-related targets (such as IL-17A, IL-5, IGF-1, CD40LG, and TAP2), thereby influencing protective immunity, NK cell function, antibody responses, and antigen presentation. Together, these mechanisms illustrate the complex regulatory networks mediated by miRNAs in *E. multilocularis* infection. This figure was created using Adobe Illustrator

In *E. multilocularis*, experimental studies have identified 44 mature miRNAs [32, 34, 63, 64] with stage-dependent expression [31, 34] implicated in key biological processes such as regeneration, nervous system development, environmental adaptation, and host modulation, as summarized in Figure 5.

Despite this extensive experimental characterization and the biological relevance of *E. multilocularis* miRNAs across developmental stages (Figure 5A), their translational investigation into human infection remains markedly limited. Based on the studies included in this systematic review, only one study reported detecting an *E. multilocularis*-derived miRNA, emu-miR-novel-1-3p, in plasma samples from patients with active AE. Though its function remains unknown, this miRNA has been proposed as a candidate biomarker based on its reported high sensitivity and specificity. Interpretation of its biological and clinical relevance is further limited by the lack of functional characterization and independent validation. This further underscores the need for translational studies before clinical implementation.

Importantly, preclinical studies have identified functionally relevant *E. multilocularis* miRNAs that may inform future translational research aimed at prioritizing candidate molecules for investigation in human biological samples. In particular, highly expressed parasite miRNAs, such as emu-miR-71 and emu-miR-4989-3p, have been suggested as potential candidates based on their abundance, involvement in host–parasite interactions, and potential participation in EV-mediated host–parasite communication through modulation of macrophage inflammatory responses and innate immune signaling pathways [65, 66], as illustrated in Figure 5B. These miRNAs have been shown to regulate parasite genes and are conserved across *Echinococcus* species, with some lacking homologs in the host miRNA repertoire.

### Expression of miRNAs in the host during CE

In studies focused on analyzing dysregulated miRNAs in patients infected with *E. granulosus s.l.* with active CE (Table 2), a constant upregulation of hsa-miR-146a-5p was observed in serum and plasma samples [49, 51]. Although this finding appears consistent across the limited available studies, the small number of investigations and variability in study design should be considered when interpreting these results.

The consistent upregulation of this miRNA underscores its potential role in immune modulation, which is particularly relevant given the low levels of pro-inflammatory cytokines, such as IL-17, reported in CE patients [49]. Hsa-miR-146a-5p is known to play a role in immune regulation, particularly through negative modulation of the IRAK1/TRAF6/NF-κB signaling pathway, as described in other infectious contexts such as *Toxoplasma gondii* and *Trypanosoma cruzi* [18]. In addition, *E. granulosus s.l* cyst fluid (EgCF) has also been shown to modulate macrophage inflammatory responses through inhibition of TRAF6 signaling [67]. Taken together, these observations are consistent with a potential role for hsa-miR-146a-5p in modulating inflammatory responses during CE. This effect may contribute to immune tolerance, potentially favoring conditions associated with chronic infection [68]. Notably, this interpretation is largely inferred from indirect or non-CE-specific evidence, and the precise role of this miRNA in human CE is still unclear.

Analysis of plasma-derived exosomes revealed a broader panel of deregulated host miRNAs in CE patients [51], supporting a potential role for EVs as mediators of host–parasite communication. The enrichment of overexpressed miRNAs belonging to families such as let-7, miR-15/16, miR-17, miR-29, miR-103/107, and miR-181, which have been associated with immune regulation, cell proliferation, apoptosis, and tissue remodeling in different biological contexts [69–72]. Other miRNAs, such as hsa-miR-132-3p, hsa-miR-93-5p, hsa-miR-335-5p, hsa-miR-101-3p, and hsa-miR-148b-3p, have also been associated with inflammatory signaling, angiogenesis, and fibrosis [73–75].

Their presence in circulating exosomes during active disease suggests that these miRNAs could participate in shaping the hepatic immune microenvironment and facilitate parasite persistence through long-range intercellular communication. Paradoxically, downregulation of host miRNAs, such as let-7 family members, miR-146, together with immuno-miRs (such as hsa-miR-155-5p, hsa-miR-223-5p, and hsa-miR-125b-5p) may reflect an impairment of host inflammatory control mechanisms, potentially contributing to immune modulation that favors parasite persistence and chronic infection [68].

Notably, both the identity and number of dysregulated miRNAs vary across studies depending on sample type and analytical platform. Exosome-based analyses generally report a broader spectrum of miRNAs compared with plasma or serum profiling, which may reflect increased sensitivity but also introduces methodological variability. This heterogeneity complicates direct comparison across studies and may hinder translation into clinical settings.

The study that evaluated the association between miRNA expression and liver fibrosis in CE showed a decrease in hsa-miR-19b-3p expression in hepatic tissue from CE patients [52]. This miRNA has been recognized as a key regulator of fibrotic processes [76, 77]. *In vitro* functional experiments using hepatic stellate cells (HSCs) further showed that reduced hsa-miR-19b-3p expression is associated with fibrogenic activation. This suppression coincides with increased expression of transforming growth factor beta receptor II (TβRII), activation of HSCs, and extracellular matrix (ECM) deposition [52].

Overall, these findings are consistent with a potential link between hsa-miR-19b-3p downregulation and TGF-β-mediated fibrotic pathways in CE, as illustrated in Figure 4C. Since much of the available evidence derives from in vitro models, the relevance of these mechanisms in human CE still requires confirmation.

Beyond the specific observations on hsa-miR-19b-3p in CE, this miRNA belongs to the miR-17-92 cluster, which has been implicated in the regulation of TGF-β1-mediated fibrosis-related pathways through targeting of TGFBRII, SMAD2, and SMAD4 [78] (Figure 4C). Therefore, loss of miR-19b expression may permit unchecked TGF-β signaling and subsequent collagen production [79].

Fibrosis plays a complex role in host immune responses to parasitic infections, including those caused by *Echinococcus* spp. While fibrosis may limit cyst expansion, it can also suppress immune cell function, much like in chronic bacterial or schistosome infections [80–82]. In CE, early pericystic fibrosis is thought to enhance host resistance by physically containing cysts [83]. However, excessive ECM production could represent a parasite-driven strategy to promote tissue adhesion, immune evasion, or even drug resistance, as has been reported in other pathogens [80, 84].

Although these observations suggest potential therapeutic implications, modulation of hsa-miR-19b-3p expression has been proposed as a potential antifibrotic strategy, and approaches such as using miRNA mimics could, in principle, influence TGF-β–mediated signaling and fibrosis-related processes. However, this hypothesis is currently supported primarily by experimental evidence, and its applicability in CE has not yet been demonstrated. Additionally, the selection of hsa-miR-19b-3p in this context appears to be based on prior knowledge of its role in fibrosis in other diseases rather than on unbiased miRNA screening approaches, which may introduce selection bias and limit the identification of CE-specific regulatory miRNAs.

To date, only a single study was identified that compared miRNA expression profiles between active and inactive cyst stages in CE [36], representing a significant gap in the current literature. The absence of independent validation limits the robustness of stage-specific miRNA signatures and the generalizability of these findings.

In this study, early active CE stages (CE1–CE3a) were associated with downregulation of hsa-miR-4659a-5p, hsa-miR-4518, hsa-miR-3977, hsa-miR-4692, hsa-miR-181b-3p, and hsa-miR-4491, while only hsa-miR-4687-5p appeared downregulated in inactive CE (CE4 and CE5)[36].

Downregulated miRNAs in early active disease were associated with pathways related to oncogenic and tumor suppressor gene expression, as well as expression of immunoglobulin superfamilies [36], whereas those downregulated in more advanced stages (e.g., CE3b) tended to affect immune response regulation and mechanisms involved in pathogen control and clearance. Some miRNAs downregulated in CE have been proposed as biomarkers for cancer diagnosis and prognosis [36, 85].

Although *Echinococcus* spp. does not induce malignant transformation, CE shares several tumor-like biological features, including infiltrative growth, angiogenesis, and extensive extracellular matrix remodeling. In this context, miRNA downregulation may reflect the activation of molecular programs that facilitate parasite persistence. Notably, previous studies have shown that overexpression of this hsa-miR-4687-5p can impair the intracellular survival of certain pathogens [86], although its role in CE remains unclear. These results suggest the existence of miRNA regulatory patterns associated with disease stage. Given that these findings derive from a single study, their clinical relevance should be interpreted cautiously.

### Expression of miRNAs in the host during AE

Studies investigating host miRNA dysregulation in AE have shown that *E. multilocularis* infection induces significant alterations in host miRNA expression profiles [53, 54]. Compared with CE, translational evidence in human AE is still considerably less developed.

In hepatic tissue samples from patients with liver alveolar echinococcosis (LAE), four differentially expressed miRNAs were identified: hsa-miR-1237-3p, hsa-miR-33b-3p, and hsa-miR-483-3p were upregulated, whereas hsa-miR-4306 was downregulated. In analyses of plasma samples from the same patient cohort, only hsa-miR-483-3p showed consistent upregulation across both hepatic tissue and plasma samples [53], which may indicate differential detectability and tissue specificity of host miRNAs depending on the biological sample. Nonetheless, the limited number of studies currently limits the relevance of these findings.

Functional insights suggest that the host miRNAs dysregulated during AE may be associated with immune-related pathways potentially involved in parasite establishment and early persistence. Collectively, these miRNAs target 137 genes involved in transcriptional regulation, liver injury responses, and immune function. For example, among the miRNAs previously identified as dysregulated in AE, hsa-miR-1237-3p has been linked to the regulation of IL-17A and IL-5 cytokines (Figure 5C), both crucial for protective Type 2 immune responses against helminths [53, 87]. Downregulation of these cytokines could influence eosinophil activation, mast cell function, and IFN-γ-mediated responses, which may contribute to an immunological environment that could favor parasite establishment.

Similarly, hsa-miR-483-3p, also upregulated in AE, has been associated with the regulation of IGF-1, a key factor for natural killer (NK) cell development and cytotoxic function [88, 89]. High expression of this miRNA may reduce NK cell-mediated defense and immune regulation (Figure 5C), although direct evidence in AE remains limited. In addition, increased hsa-miR-483-3p expression has been linked to fibrotic processes through the regulation of autophagy [90], a pathway relevant to both host defense and parasite persistence [91].

Given that hsa-miR-4306 is downregulated in LAE, increased expression of its predicted target genes may be expected, potentially influencing immune signaling pathways, as illustrated in Figure 5C. In this context, hsa-miR-4306 has been linked to the regulation of CD40 Ligand (CD40LG) and ATP Binding Cassette Subfamily B Member (TAP2), both of which are involved in key immune processes. Alterations in these pathways could affect antigen presentation and cytotoxic response [92], as well as antibody-mediated immunity, while skewing antibody profiles toward less protective IgG against *Echinococcus* spp. [93–95]. In particular, changes in TAP2 expression may influence CD8⁺ T cell cytotoxicity through impaired MHC class I/NKG2D-mediated signaling [96]. However, these mechanisms still require functional validation in AE.

Importantly, the consistent upregulation of hsa-miR-483-3p in both liver tissue and plasma samples suggests that this miRNA reflects systemic host responses to *E. multilocularis* infection and is detectable in circulation. This pattern highlights its potential relevance as a minimally invasive biomarker candidate for AE, although further validation is required.

Another study demonstrated upregulation of hsa-miR-125b-5p in both plasma and hepatic tissue samples from AE patients, indicating a consistent alteration across biological samples [54] (Table 3). In humans, the hsa-miR-125 family plays a key role in immune system development and immune modulation [19].

In parasitic and viral infections, hsa-miR-125b-5p has been associated with immune modulation and the persistence of chronic infection, respectively [97, 98]. Mechanistically, this miRNA has been shown to regulate IL-1β–induced inflammatory gene expression through targeting of TRAF6-mediated MAPK and NF-κB signaling pathways in human cells [99]. These observations suggest a potential role for hsa-miR-125b-5p in immune regulation in AE, possibly contributing to inflammation control and chronic infection. However, its relevance in AE remains to be established, as current evidence is limited and largely derived from indirect or non-AE-specific studies.

### Comparative miRNA signatures in CE and AE

Regarding comparative analyses between CE and AE (Table 4), the available evidence remains limited and suggests shared host miRNA dysregulation across echinococcosis rather than clearly distinct CE-specific and AE-specific signatures. Several miRNAs were reported to be upregulated in both diseases, including members of the hsa-miR-181 and hsa-miR-29 families, as well as hsa-miR-21-5p, hsa-miR-146a-5p, and hsa-miR-214-3p. In contrast, members of the let-7 family, along with hsa-miR-15b-5p, hsa-miR-16, hsa-miR-17-5p, miR-142-5p, and miR-125b-5p, were reported to be downregulated [55]. These shared patterns suggest that both diseases may involve common regulatory pathways related to immune tolerance and tissue remodeling. However, the current evidence remains preliminary and does not yet support assigning these liver tissue signatures exclusively to one disease form.

Another study analyzed miRNA expression profiles stratified by clinical stage and treatment status in patients with CE and AE (Table 4). When comparing patients with active AE and treated AE, distinct miRNA expression patterns were observed, suggesting dynamic regulation of host miRNAs in relation to disease activity and therapeutic intervention. In active AE, the upregulation of hsa-miR-1246 and hsa-miR-122-5p, together with the downregulation of hsa-miR-144-5p, may reflect coordinated regulatory changes affecting inflammatory pathways. While hsa-miR-1246 and hsa-miR-122-5p have been associated with pro-inflammatory functions, hsa-miR-144-5p is known to inhibit inflammatory signaling [100–103]. Additionally, hsa-miR-122-5p has been linked to liver disease through the regulation and remodeling of hepatic metabolism [103]. Overall, this expression pattern may reflect coordinated modulation of host immune responses and hepatic metabolic pathways characteristic of active AE [104], potentially linking inflammatory regulation with metabolic remodeling of the infected liver. However, these interpretations should be approached cautiously, particularly for hsa-miR-1246, which may partially reflect U2-derived RNA fragments.

In contrast, the upregulation of miRNAs such as hsa-miR-150-5p and hsa-miR-483-5p in treated AE patients may indicate a shift toward regulatory programs associated with immune rebalancing and tissue remodeling. Hsa-miR-150-5p is a well-characterized immuno-miR that regulates cell immune differentiation and function, and whose increased expression has been associated with enhanced IL-10 [105, 106]. Similarly, hsa-miR-483-5p has been reported to be dysregulated in several chronic diseases, where it is linked to immune modulation and tissue remodeling. In this context, its upregulation in treated AE patients may reflect reparative processes associated with chronic disease evolution or partial resolution following therapy [107]. Notably, the consistent upregulation of hsa-miR-122-5p and downregulation of hsa-miR-144-5p in both active and treated AE suggest that some miRNAs may reflect ongoing hepatic involvement rather than disease activity alone. However, as these observations are derived from a single study, their interpretation remains limited and speculative.

In CE, miRNA expression profiles across patient groups appeared to be relatively similar (Table 4), primarily associated with the regulation of the immune response and hepatic injury, suggesting the activation of shared host response programs across different pathological states of the disease. Differences between groups were limited to a small subset of miRNAs. Specifically, inactive CE was characterized by the downregulation of hsa-miR-1-3p, while treated CE showed selective downregulation of hsa-miR-590-3p. In contrast, NPLs were distinguished by the downregulation of hsa-miR-431-5p. The selective dysregulation of these miRNAs may reflect distinct underlying biological processes across disease states. Notably, all of these miRNAs are downregulated, suggesting an overall attenuation of the biological pathways they normally facilitate.

Hsa-miR-1-3p is widely recognized as a marker of hepatic injury [108], and its reduced expression may indicate a decrease in damage-associated signaling. In contrast, hsa-miR-590-3p is considered an onco-miR with roles in promoting cell proliferation and survival, and its downregulation in treated CE may reflect attenuation of proliferative or reparative programs following therapy [109]. Meanwhile, hsa-miR-431-5p has been linked to immune modulation in the context of infectious and inflammatory diseases [110, 111]. Its selective downregulation in non-parasitic liver lesions may point to reduced immune regulatory signaling, suggesting potential differences in inflammatory and immune pathways between parasitic and non-parasitic hepatic pathology.

In contrast to AE, where more dynamic miRNA patterns appear to be associated with disease activity and treatment status, CE profiles seem comparatively stable across clinical conditions.

Despite the currently limited evidence, the data synthesized in this review suggest that miRNA expression profiles in CE and AE primarily reflect host-driven regulatory programs that may be partially shared between both forms of the disease. This suggests that many of the identified miRNAs capture general host responses, which have important implications for their clinical interpretation.

At the same time, this shared signature represents a key limitation, as it reduces the specificity of miRNA profiles for differential diagnosis between CE and AE. While some of these miRNAs may provide insights into conserved pathogenic pathways that could inform future therapeutic research, their ability to provide parasite- or species-specific discrimination remains limited.

Nevertheless, miRNA expression profiles appear to exhibit dynamic behavior influenced by disease stage and treatment status. This may enhance their potential utility for patient stratification, disease activity monitoring, and assessment of therapeutic response, particularly when integrated with clinical, serological, and imaging parameters.

From a clinical perspective, according to the available evidence, miRNA-based approaches are unlikely to replace established diagnostic tools in echinococcosis. However, their potential utility may lie primarily in applications such as patient stratification, assessment of disease activity, monitoring of therapeutic response, and early detection of relapse, particularly in cases where conventional diagnostic tools yield inconclusive results. Parasite-derived miRNAs may provide greater etiological specificity and may be more informative for confirming active infection, whereas host-derived miRNA profiles predominantly reflect host-regulated inflammatory, fibrotic, and immune processes. As such, they may be more suitable as indicators of disease activity and treatment response rather than as standalone diagnostic markers. Given the chronic nature of cystic and alveolar echinococcosis and the dynamic host–parasite interactions that characterize these diseases, integrated miRNA signatures may provide additional insight by detecting molecular changes that precede or accompany clinical and radiological findings.

Compared to currently available diagnostic tools, miRNA-based biomarkers offer potential advantages such as minimal invasiveness and the ability to reflect dynamic host–parasite interactions. However, they also face important limitations related to specificity and overlap with other inflammatory or hepatic conditions. Therefore, their clinical value is most likely to emerge when used in combination with other diagnostic strategies. Similar considerations apply in the veterinary field, where echinococcosis is primarily monitored using imaging, fecal flotation, post-mortem inspection, and molecular and serological tools [112], reinforcing the relevance of a complementary diagnostic approach.

Within this complementary translational framework, current evidence suggests that egr-let-7-5p and egr-miR-71-5p are potential candidates for further investigation in CE, owing to their abundance, detectability in accessible biological fluids, and functional involvement in parasite–host interactions. In addition, hsa-miR-146a-5p may also represent a host-derived biomarker candidate for CE, given its consistent upregulation in plasma and serum samples from patients with active disease and its established role in immune regulation. Together, these features support their potential application in contexts requiring either etiological specificity (parasite-derived miRNAs) or assessment of disease activity (host-derived miRNAs). In AE, hsa-miR-483-3p has been proposed as a candidate biomarker due to its consistent dysregulation in both liver tissue and plasma, suggesting systemic relevance and feasibility for minimally invasive detection.

However, these observations remain preliminary, as they are based on a limited and heterogeneous body of evidence derived largely from exploratory and preclinical studies, with limited functional validation in clinical settings. Further refinement and validation will be required before these miRNA candidates can be meaningfully translated into routine clinical applications. Overall, while current evidence supports the biological and translational relevance of miRNAs in echinococcosis, the field remains at an early stage, characterized by methodological heterogeneity, limited sample sizes, and a lack of standardized validation approaches.

Beyond the limitations discussed above, important methodological constraints should also be considered. The overall quality of the included studies is affected by substantial heterogeneity, the widespread use of case–control designs, and the absence of robust diagnostic validation frameworks. These characteristics may introduce spectrum bias and potentially overestimate diagnostic performance, thereby limiting the generalizability of the findings to real-world clinical settings. Furthermore, many studies have focused on exploratory or mechanistic analyses without reporting standardized diagnostic accuracy metrics or including independent validation cohorts. This reduces the strength of the evidence supporting the clinical applicability of the proposed miRNA biomarkers. Consequently, while current findings highlight the potential relevance of miRNAs in echinococcosis, the existing evidence base remains insufficient to support clinical implementation. Well-designed prospective studies will be required to establish their true diagnostic value.

Most excluded studies were based on in vitro or animal models, reflecting the current predominance of experimental research focused on host–parasite interactions and miRNA-related mechanisms. This trend highlights a significant gap between experimental findings and human clinical studies, limiting the translational relevance of the available evidence and underscoring the need for more robust clinically oriented investigations.

### Limitations and future directions

An important limitation of this systematic review relates to the search strategy and the evolving taxonomy of the *E. granulosus s.l.* complex. Although “*E. granulosus*” was used as a primary search term, studies using updated species nomenclature (e.g., *E. canadensis*) were also retrieved during the screening process. Nevertheless, variability in taxonomic terminology across the literature may have affected the comprehensiveness of study retrieval.

Another important limitation concerns the marked heterogeneity in miRNA detection and quantification methodologies across the included studies. The platforms employed ranged from high-throughput miRNA-seq to microarrays to more targeted techniques such as qPCR. This methodological heterogeneity not only affects the sensitivity and specificity of miRNA detection but also compromises the reproducibility of results and limits the ability to perform direct comparisons across studies. Certain miRNAs that are detectable through next-generation sequencing platforms may go undetected when using less sensitive techniques such as microarrays or qPCR, leading to discrepancies in the reported expression profiles.

Additionally, the lack of standardization in data normalization (the use of different endogenous controls or bioinformatics strategies) adds a layer of variability that could affect the interpretation of miRNA profiles associated with *Echinococcus* spp. infections. This heterogeneity may introduce bias in the synthesis of results and underscores the need to establish standardized criteria for future miRNA expression studies in the context of echinococcosis, particularly if the goal is to validate consistent and clinically applicable diagnostic or therapeutic biomarkers.

The overall risk of bias across the included studies should be considered. The predominance of case–control designs and the lack of independent validation cohorts may lead to overestimation of biomarker performance and limit the generalizability of the findings.

A further limitation is that none of the clinical studies included determined the infecting *Echinococcus* species, with patients generally classified as having AC or CE without species-level confirmation. This lack of taxonomic resolution may reduce the specificity of reported miRNA signatures and hinder the identification of species-specific biomarkers. In turn, this limitation may affect the interpretability and potential clinical applicability of these findings, particularly in distinguishing infections caused by different species within the *E. granulosus s.l*. complex

Although the number of cross-sectional studies has increased, there is a notable lack of prospective investigations assessing how miRNA expression evolves during antiparasitic therapy or following surgical intervention, which hinders the understanding of whether miRNAs can be proposed as biomarkers for monitoring disease progression, therapeutic response, or detecting recurrences. Implementing prospective and longitudinal studies, including sampling at different time points during treatment and clinical follow-up, would be relevant to validate their clinical utility and establish their temporal behavior in real-life settings. This type of study represents a key opportunity to advance the translational use of miRNAs in the management of echinococcosis.

The majority of the included studies focused exclusively on individuals with echinococcosis, without including control groups affected by other hepatic or parasitic diseases. This limitation prevents a rigorous assessment of the diagnostic specificity of the identified miRNA signatures and may compromise their clinical applicability, particularly in regions where multiple helminth infections coexist, increasing the risk of false positives and overdiagnosis of echinococcosis.

### Challenges for the clinical translation of miRNA-based therapies

Despite the growing interest in miRNA-based diagnostics and therapeutics, several biological and translational challenges remain before miRNA-based approaches can be implemented in the clinical management of echinococcosis.

Most identified host-derived miRNAs reflect general biological processes, including inflammation, fibrosis, and tissue remodeling, rather than infection-specific signals. This limits their specificity for independent etiological diagnosis and increases the risk of overlap with other liver or parasitic diseases, in which some of these miRNAs are dysregulated [18, 19, 113, 114].

From a therapeutic perspective, miRNA-based interventions face several challenges. These include the need for efficient and tissue-specific delivery systems, the pleiotropic nature of miRNA targets, and the risk of unintended off-target effects that may disrupt essential host pathways [115, 116]. A particularly relevant concern in miRNA-based treatment of echinococcosis is that several parasite-derived miRNAs share highly conserved seed sequences with host miRNAs [34, 117]. This raises the possibility of cross-reactivity and unintended modulation of host gene networks when designing miRNA-based therapies. This challenge is further compounded by the context-dependent functions of many miRNAs, whose effects may vary according to tissue type, disease stage, and host immune status [118, 119], thereby increasing the risk of unpredictable biological outcomes. Furthermore, defining optimal dosing regimens that achieve therapeutic efficacy while minimizing toxicity remains a major obstacle for the clinical translation of miRNA-based therapies, particularly given their pleiotropic effects and potential accumulation in non-target tissues [116].

Beyond biological and technical limitations, several practical and regulatory considerations must be addressed before miRNA-based approaches can be incorporated into routine clinical practice. These include the standardization of analytical pipelines, validation in large multicenter and longitudinal cohorts, cost-effectiveness assessments, and the definition of stepwise implementation strategies (rollout plans) that facilitate their integration into existing diagnostic and therapeutic workflows.

At the methodological level, additional challenges remain in the use of miRNAs as biomarkers. These include variability in sample collection procedures, choice of profiling platforms, sample volume, and RNA extraction protocols. Together, these factors represent important obstacles to standardization and reproducibility across clinical settings [120].

In parallel, regulatory and safety requirements represent major barriers to clinical translation. Despite growing interest in their therapeutic potential, regulatory agencies such as the US Food and Drug Administration (FDA) and European Medicines Agency (EMA) require robust evidence of safety, efficacy, and specificity. This requirement is particularly challenging given the pleiotropic nature of miRNAs and the inherent risk of off-target effects [116]. Furthermore, available delivery systems introduce an additional layer of regulatory complexity. These systems must demonstrate not only efficient and tissue-specific delivery but also acceptable profiles of immunogenicity, genotoxicity, biodistribution, and pharmacokinetics. These requirements have been the primary limiting factors in the progression of many miRNA-based strategies to advanced clinical trials [116].

Additional regulatory and ethical considerations are especially relevant in echinococcosis-endemic regions, where limited resources and restricted access to advanced molecular diagnostics may hinder implementation. Clinical translation of miRNA-based approaches will require standardized analytical procedures and validation in large, geographically diverse cohorts. Ethical challenges include ensuring equitable access to molecular diagnostics and minimizing the risk of overdiagnosis or misclassification in populations exposed to multiple parasitic infections, where overlapping miRNA signatures can complicate interpretation.

Collectively, these limitations underscore that, despite promising preclinical advances, the clinical implementation of miRNA-based therapies remains limited and will require significant methodological, regulatory, and technological efforts, particularly in endemic areas. Furthermore, the chronic nature of echinococcosis and stage-dependent disease dynamics must be carefully considered before such strategies can be deemed viable in routine clinical practice.

## Conclusions

Echinococcosis is a parasitic disease caused by cestodes of the genus Echinococcus. In humans, *E. granulosus s.l.* and *E. multilocularis* are responsible for CE and AE, respectively, which are the most common and clinically significant forms of the disease. Like other helminths, *Echinococcus* spp. modulate the host immune response through various mechanisms, including the dysregulation of miRNAs. In the parasite, miRNAs regulate key processes including development, metabolism, invasion, and virulence. In the host, dysregulated miRNA-mediated signaling pathways may result in immune activation or suppression, promoting parasite clearance or facilitating infection, respectively.

Although evidence from patient-based studies remains limited, available data suggest that miRNAs may serve as complementary biomarkers for diagnosis, patient stratification, prognosis, disease monitoring, and assessment of therapeutic response. However, the current body of evidence is constrained by important methodological limitations, including the predominance of case–control designs, small sample sizes, and the lack of standardized diagnostic validation. These factors may lead to an overestimation of diagnostic performance and limit the generalizability of findings to clinical practice.

Therefore, while miRNAs show translational potential in echinococcosis research, their clinical utility remains to be established. Further validation in well-designed prospective studies with standardized methodologies and clinically relevant populations is required before their implementation in clinical settings. In addition, they may inform future miRNA-based therapeutic research in echinococcosis.

## Data Availability

The data supporting the conclusions of this article are included within the article.

## Abbreviations

miRNA(s): MicroRNA(s)
CE: Cystic echinococcosis
AE: Alveolar echinococcosis
PSCs: Protoscoleces
WHO: World Health Organization
EVs: Extracellular vesicles
MAPK: Mitogen-activated protein kinase
LAE: Liver alveolar echinococcosis
NPLs: Non-parasitic liver lesions
TGF-β: Transforming growth factor beta
RT-PCR: Reverse transcription polymerase chain reaction
miRNA-seq: MicroRNA sequencing
sRNA-seq: Small RNA sequencing
TβRII: Transforming growth factor beta receptor II
ECM: Extracellular matrix
EgCF: *Echinococcus* *granulosus* Cyst fluid
HSCs: Hepatic stellate cells
IGF-1: Insulin-like growth factor 1
IFN-γ: Interferon gamma
IL-17A: Interleukin 17ACD40 ligand
TAP2: Transporter associated with antigen processing 2
IRAK1: Interleukin-1 receptor-associated kinase 1
LPS: Lipopolysaccharide
NF-κB: Nuclear factor kappa B
EMA: European Medicines Agency
FDA: U.S. Food and Drug Administration
